# Patterns and Factors Associated with the Length of Hospital Stay in the Psychiatry Department of a Tertiary Care Teaching Hospital in Nepal: An Observational Study

**DOI:** 10.31729/jnma.v63i292.9275

**Published:** 2025-12-31

**Authors:** Sulochana Joshi, Mankaji Thapa, Anusha Manandhar, Rabi Shakya

**Affiliations:** 1 Department of Psychiatry, Patan Academy of Health Sciences, Lagankhel, Lalitpur, Nepal; 2 Gorkha Hospital, Gorkha, Nepal; 3 Trishuli Hospital, Nuwakot, Nepal

**Keywords:** *electroconvulsive therapy*, *inpatients*, *length of stay*, *mental health services*, *therapeutics*

## Abstract

**Introduction:**

Inpatient treatment in psychiatry is important due to several reasons, including stigma, cost, family support, admission indication, as well as availability of beds and other resources. In our setting, limited bed availability and challenges in inpatient services hinder the delivery of quality patient care. Therefore, information on the awareness, functionality, and needs of the inpatient services at our centre is imperative. This study aims to explore the clinicodemographic profile of psychiatric inpatients and examine their association with the length of hospital stay.

**Methods:**

This was a retrospective cohort study of all inpatients from June 2013 to May 2017. The clinicodemographic variables were recorded as per the developed proforma. We summarized the sociodemographic and other characteristics using median and proportions. To identify the factors with a significant impact on the length of stay, we performed multiple linear regression analyses.

**Results:**

Of 1085 psychiatric inpatient records available, 1034 were included. The median length of stay was 8 days (Interquartile Range, 5 to 12 days). The patients with psychotic disorders and those receiving electroconvulsive therapy were more likely to have a longer length of stay by 30% and 146%, respectively.

**Conclusions:**

The overall length of stay was relatively short. However, the presence of psychotic disorders and the use of electroconvulsive therapy were associated with prolonged hospitalization.

## INTRODUCTION

Inpatient treatment services have been increasingly available in Nepal, including in general hospitals, which has contributed to a gradual de-stigmatization of these services and heightened public awareness.^1^ However, the limited availability of beds is an important challenge faced by the institutions, which is further compounded by the often prolonged length of hospital stay among the psychiatric inpatients. Besides, the length of stay (LOS) for patients is influenced by factors beyond clinical needs, such as cultural aspects.^2^

Several factors impacting the LOS have been identified, indicating different aspects of illness such as psychopathology, predominance, service utilization, and preventive factors.^3^ Understanding these factors can help improve inpatient services by identifying what influences the length of stay, thereby guiding potential interventions to reduce it.

This study aims to explore the clinicodemographic profile of inpatients and examine the factors associated with the LOS.

## METHODS

This is a retrospective cohort study conducted by reviewing the medical records of all inpatients in the Department of Psychiatry at Patan Academy of Health Sciences (PAHS), a tertiary care teaching hospital, over 4 years from June 2013 to May 2017. Using hospital numbers from the inpatient register in the Department of Psychiatry and the hospital medical record section, we retrieved the relevant data. We recorded clinical and demographic information (age, gender, marital status, diagnosis spectrum, comorbidity, ECT, readmission, and residence) according to a predefined data collection tool. The Institutional Review Committee (IRC) of PAHS (Ref-drs1803111168) granted ethical approval and waived the requirement for individual informed consent, as the study involved no direct patient interaction or intervention. To protect confidentiality and privacy, we anonymized all data by replacing each hospital number with a unique identifier (LOS1, LOS2, ...) and removing names and other personal identifiers before analysis. We entered the data in Microsoft Excel (Microsoft Office 365, Microsoft Corporation, Washington, United States) and performed the analysis using R Satirical software (R Core Team. R: A language and environment for statistical computing. Vienna, Austria). We summarized the clinical and other characteristics using the median (Interquartile Range [IQR]) and proportions. We performed multiple linear regression analyses to identify the factors with a significant impact on the LOS. The initial set of independent variables included age, sex, marital status, diagnosis spectrum, comorbidity, ECT, readmission, and residence. We regrouped the related diagnostic categories into broader, clinically meaningful groups to improve the precision of estimates and enhance the interpretability of the model without compromising the clinical relevance of the variable. Using an Allen-Cady modified, backward elimination procedure for variable selection to minimize the risk of Type I error, we iteratively removed variables with the higher p-values while retaining age and sex a priori as potential confounders based on clinical relevance. We checked the assumptions of the multiple linear regression model obtained and took appropriate measures when the assumptions were unmet. We examined the collinearity between the variables in the model with the Variance Inflation Factor (VIF) and regarded that multicollinearity is present for the variables whose VIF exceeded 10. For the final model, we also examined the potential interactions between the independent variables in the model. All the significance tests were two-sided, with a p-value <0.05 considered significant.

## RESULTS

A total of 1085 psychiatric inpatient records were reviewed, and 1034 were included in the study after omitting the duplicate and incomplete records of 51 patients. Most of the patients belonged to the 25 to 64 years age group 702 (67.89%), were males 655 (63.35%), married 846 (81.82%), and resided outside Kathmandu valley 459 (44.39%). The median age was 33 (IQR 24 to 45) years. Substance use disorder was the most common diagnosis. For most patients, this was their index psychiatric admission. The median hospital stay duration was 8 (IQR 5 to 12) days. Most of the patients improved at the time of discharge (Table 1).

**Figure 1:**
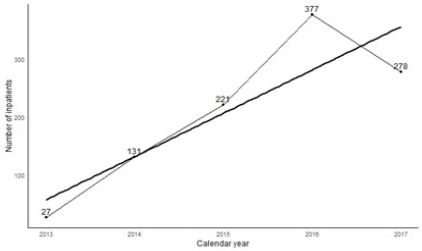
Trends of Hospital admissions admitted via emergency.

The admissions were both voluntary and involuntary, admitted by OPD, emergency, and consultation liaison. Common indications for admissions were self-harm, catatonia, aggressive behavior, withdrawal states, requirement of ECT, and diagnostic clarification. Among these, most patients with self-harm and catatonia were admitted via emergency. The number of psychiatric admissions increased yearly. The sharp increase in admissions between 2013 and 2014 (from 27 to 131) paralleled the increase in bed capacity from 6 to 10 beds (Figure 1).

**Figure 2:**
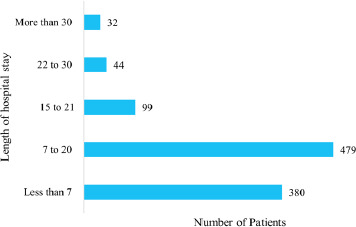
Patterns of length of hospital stay (days).

The LOS ranged from one day to 67 days. The median LOS was 8 (IQR 5 to 12) days. There were 32 (3.09%) patients who stayed more than a month (Figure 2).

**Table 1:** Baseline sociodemographic variables (n= 1034).

Characteriftics	n(%)
**Age**	
0 - 14 Years	32(3.09)
15 - 24 Years	246(23.79)
25 - 64 years	702(67.89)
65 years and above	54(5.22)
**Sex**	
Male	655(63.35)
Female	379(36.65)
**Marital Status**	
Married	846(81.82)
Single	188(18.18)
**Residence**	
Out of Kathmandu valley	459(44.39)
Lalitpur	349(33.75)
Kathmandu	195(18.86)
Bhaktapur	3 (3.00)
**Diagnosis spectrum**	
Substance use disorders	378(36.56)
Mood disorder	241(23.32)
Psychotic disorders	203(19.63)
Neurotic, stress-related, and somatoform disorders	120(11.61)
Deliberate self-harm	32(3.09)
Organic mental disorders	31(3.00)
Epilepsy	16(1.55)
Mental retardation	7(0.68)
Personality disorders	6(0.58)
**Comorbidity**	
Medical	736(71.18)
Psychiatric	162(15.67)
None	91(8.80)
Both medical and psychiatric	45(4.35)
**Readmission**	
First admission	908(87.81)
Readmission	126(12.19)
**Electroconvulsive therapy**	
Not received	1003(97.00)
Received	31(3.00)
**Outcome at discharge**	
Improved	940(90.91)
Discharge on request	76(7.35)
Leave against medical advice	11(1.06)
Transferred	7(0.68)

**Table 2:** Multivariable linear regression of factors associated with log-transformed length of stay (n = 1034).

Factor	ß (coefficient)	SE	exp(ß)	Change in LOS	95% CI for change in LOS	p-value
Intercept	1.653	0.1	6.203	-	-	-
**Age group (years)**						
0-14 (Ref.)	-	-	-	-	-	-
15-24	0.200	0.1	1.22	22.10%	-4.9% to 56.8%	0.196
25-64	0.220	0.1	1.25	24.60%	-2.4% to 59.0%	0.163
≥ 65	0.261	0.2	1.30	29.80%	-3.5% to 74.5%	0.215
**Sex**						
Female (Ref.)	-	-	-	-	-	-
Male	0.034	0.1	1.03	3.50%	-5.8% to 13.7%	0.639
**Diagnosis spectrum**						
Non-psychosis (Ref.)	-	-	-	-	-	-
Psychosis	0.264	0.1	1.30	30.20%	18.4% to 43.1%	< 0.001
**ECT treatment**						
No (Ref.)	-	-	-	-	-	-
Yes	0.902	0.1	2.46	145.50%	93.3% to 214.6%	< 0.001

LOS = Length of stay, SE = Standard Error

From the initial multiple linear regression model with the variables age, sex, marital status, diagnosis spectrum, comorbidity, ECT, readmission, and residence, we obtained a parsimonious model with age, sex, diagnosis spectrum, and ECT after Allen-Cady modified backward elimination strategy of variable selection. Age was non-linear in its association with LOS in component-plus-residual plots; it was therefore categorized into four groups (Table 2). The distribution of LOS was markedly right-skewed, and diagnostic evaluation demonstrated violations of the normality and homoscedasticity assumptions; therefore, we performed a log-transformation of LOS (log-LOS), which substantially improved the distribution of residuals and removed heteroscedasticity, which we reported as the final model. VIFs were <10 for all predictors, indicating no evidence of problematic collinearity. The study design, which included individual patient-level observations without repeated measures, supported the assumption of independent observations. We evaluated the potential interaction terms between age group, sex, diagnosis spectrum, and ECT and found no statistically significant interactions. For interpretability, we exponentiated the coefficients from the log-transformed model and expressed them as a percentage change in LOS. We found that the Psychotic diagnosis (p < 0.001) and receiving ECT (p < 0.001) had statistically significant associations with the LOS among the psychiatric inpatients. Patients who had psychosis had an estimated 30% longer length of stay (β = 0.264; 95% CI 18.4 - 43.1) on average compared to the patients who had non-psychotic diagnoses. Similarly, patients who received ECT had a 145.5% longer length of stay (P = 0.902; 95% CI: 93.3 - 214.6) compared with those who did not receive ECT (Table 2).

## DISCUSSION

Our study aimed to assess the patterns and factors associated with the LOS among psychiatric admissions in a tertiary care center in Nepal. We found that the psychiatric inpatients were mostly young married males residing outside Kathmandu Valley. Substance use disorders, mood disorders, and psychotic disorders accounted for most admissions. Patients who had psychotic diagnoses and those receiving ECT had longer hospital stays as compared to those who did not.

The median LOS was 8 (IQR 5 to 12) days. This finding is similar to the studies done in central Nepal with a hospital stay of 7-14 days.^4,5^ However, our finding was in contrast to studies done in Italy, eastern Nepal, Brazil, Ethiopia, and Nigeria, which had longer stays of 13.3 days, 19.4 days, 20.5 days, 22 days, and 28.7 days, respectively.^1,6-9^ Few patients (3.09%) stayed more than a month, which is alike the study by Dhungana et al (1%) and Shrestha et al (6.69%) but different from that of Basnet et al (18.2%).^4-6^ The reason for shorter LOS in our study could be clinical improvement achieved in most of the patients, which further could be due to the enrollment of mostly substance use disorder patients in an acute state.^10^

The majority of patients were admitted for substance use disorder (36.56%) in our study, whereas studies done in Kathmandu Valley, Nepal, and Israel had psychotic disorders as the most common diagnosis.^4, 5-11^ Similarly, mood disorders were the most common diagnosis of the inpatients in the study done in eastern Nepal.^6^ Psychotic and mood disorders were the common causes for admission in the study done in Nigeria.^7^ The reason for this difference in our study could be the good and robust consultation liaison psychiatry and the presence of medical comorbidity in patients with substance use disorders. Also, the availability of a government psychiatry hospital nearby could have decreased the volume of patients with mood and psychotic diagnoses in our center, hence making it the second and third most common diagnoses in our inpatients.Patients with a diagnosis of a Psychotic disorder and receiving ECT treatment were associated with longer stay in our study, which is similar to many studies.^1-3,7, 8, 11-16^

Our patients with psychosis had 30% longer stay on average than the patients with non-psychotic disorders. The longer LOS in psychosis could be accounted for by the chronic nature, complex symptomatology, difficulty in treating acute symptoms, and negative symptoms of psychosis.^13^ Also, a study done by Tulloch et.al. in the UK found an association between longer LOS with the severity of illness and the need for rehousing.^15^ This illness severity could be the cause instead of a psychotic diagnosis alone in our study, too, which could not be assessed in the current study.

In our study, only 31 patients (3%) received ECT, and the use of ECT was associated with longer LOS, which was comparable to other studies.^3, 7-17, 18^ The reasons could be severe and treatment-resistant cases and complications of ECT, prolonging the hospital stay.^17, 19^ Our patients who received ECT had 145.5% longer stay than those who did not, which is different from that reported in the study by Draper et al.^19^ This difference could be due to the difference in methodology and study population, as it included geriatric patients only and consisted mostly of major depression and delirium.

The majority of patients had medical comorbidity (71.18%). This finding is in contrast to a study by Basnet et al, which reported few comorbidities.^6^ This reason could be that most of our patients had substance use disorder and comorbidity as well. The presence of comorbidity was not associated with LOS in our study, which is similar to a few studies.^7, 19^ but different than other studies.^20, 21^ The presence of comorbidities requires consultation and treatment from other specialties which eventually prolong the LOS.^3^ The reason comorbidities did not affect LOS in our study could be the small sample size, missing data and mild physical comorbid illness, underdiagnosis due to lack of proper documentation which has less impact on course of illness.

The majority of patients were admitted once (87.81%) and readmission was minimal (12.19%) which is similar to the study by Basnet et al.^6^ The previous admission was associated with a longer stay in a few studies.^9, 11^ The readmission of patients was not associated with LOS in our study which is in contrast with many studies.^2, 13, 14, 22, 23^ The readmitted patients tend to be chronic with a tendency to have treatment resistance hence resulting in longer LOS.^3^ The readmission did not have an effect on LOS which could be attributed to a small sample of readmitted patients in our study.

The majority (67.89%) of our patients belonged to the 25 to 64 age group which is similar to the previous studies done in psychiatric inpatients in Nepal.^4-6^ The reason could be that the study was done in an adult psychiatry ward. Also, this age group is vulnerable to many psychiatric illnesses and is likely to be exposed to different stressors. Age is not found to have statistically significant association with LOS in our study which is consistent with other studies.^13, 14^ However there are studies with variable association of age with LOS which could be related to social support and comorbid illness.^11, 22, 24-26^ The reason for age not being associated with LOS in our study could be the limited sample size of the young and elderly population.

Most of our patients were male (63.35%). A high preponderance of male patients were admitted in studies from Nepal, similar to ours.^4-6^ Gender was not found to have statistically significant association with LOS in our study which is similar to other studies.^13, 14, 17^ Few studies have shown that the female gender is associated with a longer stays.^24, 25, 27^ The reason for longer LOS in females in those studies could be attributed to females having more severe depression, a higher prevalence of depression in females, and the tendency of females to get rid of daily duties.^27^ A study with more male patients and a diagnosis of Schizophrenia was also associated with longer LOS.^12^ We need to explore further the association of gender with LOS, disorders, and severity in our context.

The majority of patients were married (81.82%) in our study which is similar to the study by Shrestha et al from Nepal.^5^ The reason could be cultural, as marriage is believed to be one of the treatments for psychiatric illnesses. Also, the family acts as a strong social support which could have sought treatment for the patients. The marital status was not found to have statistically significant association with LOS in our study which is consistent with other studies.^12-14^ However, in a few studies, LOS was found to be shorter in married patients which could be explained by better social support at home facilitating earlier discharge.^2, 27^

The majority of patients were out of Kathmandu Valley (44.39%) in our study whereas previous studies from Nepal had admitted patients from nearby the center.^4-6^ This is different from previous studies of Nepal. The reason could be the popularity of our hospital as the tertiary care center as well as the availability of different other centers for those within the Kathmandu valley. The residence was not found to have statistically significant association with LOS in our study, unlike a study from Nigeria.^7^

This study benefits from a large, real-world dataset collected over four years, with systematically recorded clinical and demographic information. The use of multivariable analysis strengthens the identification of independent factors instuencing length of stay, accounting for confounders. Overall, this study captures the formative phase of our psychiatry department’s establishment, providing practical insights for improving psychiatric inpatient services in a resource-limited setting.

This study is a retrospective chart review, and only those variables that were accessible were studied. Other variables could affect LOS, like employment status, presence of children at home, type of admission, and use of restraints, and could be studied by prospective study designs. There was missing and unclear information in the chart, as there was no provision for an electronic health record system. The psychiatric comorbidities were reported in only 15.67% of the study population, which may represent under-documentation, as many comorbid psychiatric illnesses are commonly missed in routine clinical practice.^28^ The effect of comorbidity may not have been seen in this study due to missing data. The severity of illness, instead of diagnosis alone, is associated with length of stay, but the severity of the illness was not taken into consideration in this study. This study was based on patients from a single center and has, therefore, limited generalizability of the findings.

## CONCLUSIONS

Hospital stay is an expensive but important component of psychiatric services. Diagnosis of psychotic disorders and those receiving ECT were found to be associated with length of stay. The knowledge of hospital length of stay and the associated factors will provide a measure of the needs for inpatient services and aid in the allocation of resources.

## Data Availability

The data are available from the corresponding author upon reasonable request.
